# Morphometric analyses in patients treated with subthreshold laser photocoagulation for central serous chorioretinopathy

**DOI:** 10.1186/s12886-022-02732-0

**Published:** 2022-12-22

**Authors:** C. Enders, G. E. Lang, B. Mayer, J. U. Werner

**Affiliations:** Ulm University, Ulm, Germany

**Keywords:** Central serous chorioretinoparhy, subthreshold lasercoagulation, optical coherence tomography

## Abstract

**Background and objective:**

To analyze changes in selected parameters in optical coherence tomography (OCT) after subthreshold laser coagulation (ST-LP) in patients with central serous chorioretinopathy (CSCR).

**Materials and methods:**

Fifty-four eyes of 49 patients with CSCR were included in the study. Each patient underwent therapy with ST-LP with a frequency-doubled Neodym-YAG Laser and OCT imaging. In OCT the thickness of the central subfield, cube volume, average cube thickness, volume under the ETDRS grid, and average thickness under the ETDRS grid were collected.

**Results:**

Decreases in total OCT volume and central retinal subfield thickness were statistically significant (*p* < 0.05). Possible correlations were observed between visual acuity at V3 (3 months after ST-LP) and Baseline and between central retinal subfield thickness at V1 (4 weeks after ST-LP) and visual acuity at BL.

**Conclusion:**

A decrease in retinal thickness and retinal volume could be shown after ST-LP. Central retinal subfield thickness measured by OCT could be a more sensitive measure than mean retinal thickness or macular volume for early detection of disease recurrence occurring in some patients 3 months after ST-LP.

## Background and objective

Patients with central serous chorioretinopathy (CSCR) develop a serous detachment of the central neurosensory retina (NR) or an epithelial detachment of the pigment epithelium (PED). The etiology of CSCR is multifactorial and the underlying pathogenesis is not fully understood. However, circulatory dysfunction in the choroid appears to be the main cause. The choroidal vessels are probably no longer able to maintain homeostasis, leading to congestion in Haller's vascular layer and compression of the vessels of the inner choroid, with subsequent PED [1–3]. Additionally, a breakdown of the outer blood-retina barrier at the level of the RPE occurs, which leads to an accumulation of subretinal fluid (SRF) [1, 2, 4]. This extravasation of the inner choroidal layers can be seen during indocyanine green angiography (ICGA) of affected eyes [5, 6]. Hyperpermeability seems to be a crucial factor for the pathogenesis of CSCR. It has been shown that after disappearance of the leakage point in fluoresceine angiography (FA), hyperpermeability of the choroid persists in ICGA and recurrence of CSCR with a new leakage point in FA occurs in the area of hyperpermeable choroid [7, 8]. Whether this increased permeability is a result of inflammatory activity, ischemia, or stasis of the choroidal circulation remains unclear [4]. Damage to the RPE, as seen to a marked degree in the chronic form, in turn impedes the pumping function of the RPE with resorption of SRF [9].

Besides the detachment of the NR, multiple changes of the retinal structure can be found in optical coherence tomography angiography (OCT). To understand the role of OCT changes regarding diagnosis, prognosis and treatment planning in patients with CSCR, we performed further analyses based on previously published results [10]. In particular, we investigated the influence of subthreshold laser photocoagulation (ST-LP) with a Nd:YAG continuous wave laser with a wavelength of 532 nm on selected parameters in OCT. Additionally, we analyzed whether there are correlations between these parameters in OCT and with best corrected visual acuity (BCVA) before and after ST-LP.

## Patients and methods

This retrospective study was approved by the local ethic committee of Ulm University (application number 395/15) and conducted in accordance with the Declaration of Helsinki. The study plan has been published previously [10].

We recruited all consecutive patients who presented with CSCR in the outpatient clinic of a university eye clinic from 2009 to 2014, that underwent ST-LP.

The prerequisites for performing ST-LP, hence inclusion criteria for this study, were a confirmed diagnosis of CSCR with macular involvement, SRF that had not resorbed for more than 4 months or recurrence of CSCR, and evidence of at least one source point typical for CSCR in FA which was accessible to ST-LP (500 µm away from the fovea centralis and one optic disc diameter away from the optic nerve). Patients in whom OCT image quality was not sufficient for follow-up were excluded from the study. Choroidal neovascularization was excluded by fluorescein angiography and indocyanine green angiography. Further exclusion criteria were clinically relevant comorbidities with visual relevance at both anterior and posterior segment as well as recent eye surgery.

After FA and fundus photography were performed and indication to ST-LP was established, ST-LP was performed by always the same retina expert using a frequency-doubled Nd:YAG continuous wave laser with a wavelength of 532 nm (until July 2011: VISULAS 532, from July 2011: VISULAS 532 s model from Zeiss (Carl Zeiss Meditec AG, Jena, Germany). The source points determined in the FA were the target of the laser treatment. To perform ST-LP, FA-image was chosen, and the source point(s) were marked. The duration of a laser pulse was 100 ms, the spot size was 100 µm, resulting in an effective spot size of 105 µm taking contact lens correction factor into account. The laser power was titrated starting with 60 mW and increasing by 10 mW each time in such a way that in the slit lamp illumination only a very slight white coloration of the retina was visible in the laser area and a very subtle edema of the retina in the red-free light. The number of laser foci required depended on the absorption behavior of the retinal pigment epithelium. The laser foci were applied through a contact lens Mainster Focal/Grid Laser Lens (Ocular Instruments, USA) coupled by a methylcellulose gel. This treatment protocol has been published before [10].

BCVA was collected immediately before ST-LP (baseline (BL)). The further follow-up examinations were performed after 4 (visit (V1)) 8 (V2), and 12 (V3) weeks including a complete ophthalmologic examination of the anterior and posterior segments, OCT, update of medical history update and BCVA.

### Visual acuity

To determine BCVA, an autorefraction was performed first. Visual acuity was obtained at a distance of 5 m as best-corrected visual acuity using common techniques, e.g., the cross-cylinder method.

### OCT

All images included in the OCT analysis were taken with the Zeiss Cirrus 4000 and Zeiss Cirrus 5000 (Carl Zeiss Meditec Inc., Dublin, USA) with a side length of the x- and y-scan axis of 3 mm, 128 horizontal lines were acquired. Each line consisted of 512 individual A-scans.

### Analysis of thickness and volume in the OCT data set

Also before the ST-LP and at each visit with OCT analysis, the measured values of thickness of the central subfield, cube volume, average cube thickness, volume under the ETDRS grid, and average thickness under the ETDRS grid determined by Zeiss Forum were collected. The calculations were performed by Zeiss Forum in the Retina Workplace module, "Macular Thickness Analysis".

### Statistical analysis

All data were collected in Microsoft Excel 2013. Further statistical analysis was performed using SAS software (www.sas.com, version 9.4).

For inferential statistical analyses, a one-factor ANOVA was applied. The test for correlations of various parameters was performed using the Spearman rank correlation or its corresponding *p*-value. The exploratory significance level was set at *p* < 0.05 for all analyses.

## Results

Fifty-four eyes of 49 patients were analyzed. The median age was 47 years and 89% were male (48 male patients, 6 female patients), the interquartile range was 10 years. 69% of patients had a recurrence, and another 11% had persistence of SRF ≥ 6 months. A prior treatment with one or two ST-LPs or photodynamic therapy was performed in 19% of patients. The median time from last symptom onset to ST-LP was 9 weeks.

**Fig. 1:**
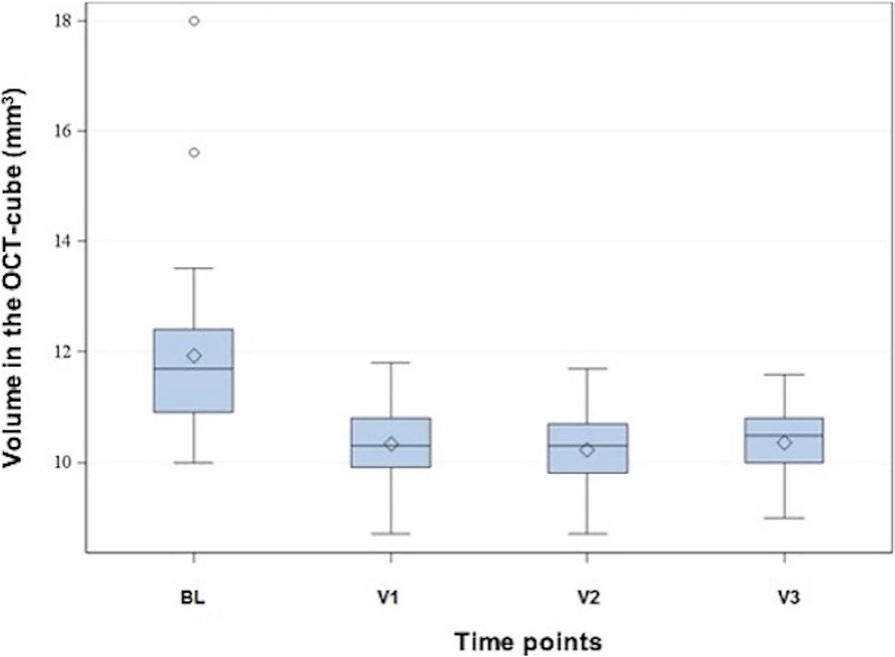
Volume in the OCT cube at the different time points. The ordinate shows the volume of the entire OCT cube (mm^3^), the abscissa reveals the different time points. The diamond indicates the mean, the horizontal line the median of the volume. The gray box in the Tukey diagram marks the range of values from the first to the third quartile, the whiskers have a length of 2 times the interquartile range. Measured values outside of this are shown by means of a circle as outliers in the diagram. OCT: optical coherence tomography; BL: baseline; V: visit

The volume of the total OCT scan at BL and at the time points V1 to V3 were measured (Fig. 1). The median volume decreased from 11.7 mm^3^ at BL to 10.3 mm^3^ at V1. This volume remained roughly constant at 10.3 mm^3^ at V2 and 10.5 mm^3^ at V3. The differences in volume were statistically significant at time points V1, V2 and V3 compared to BL (*p* < 0.05).

**Fig. 2:**
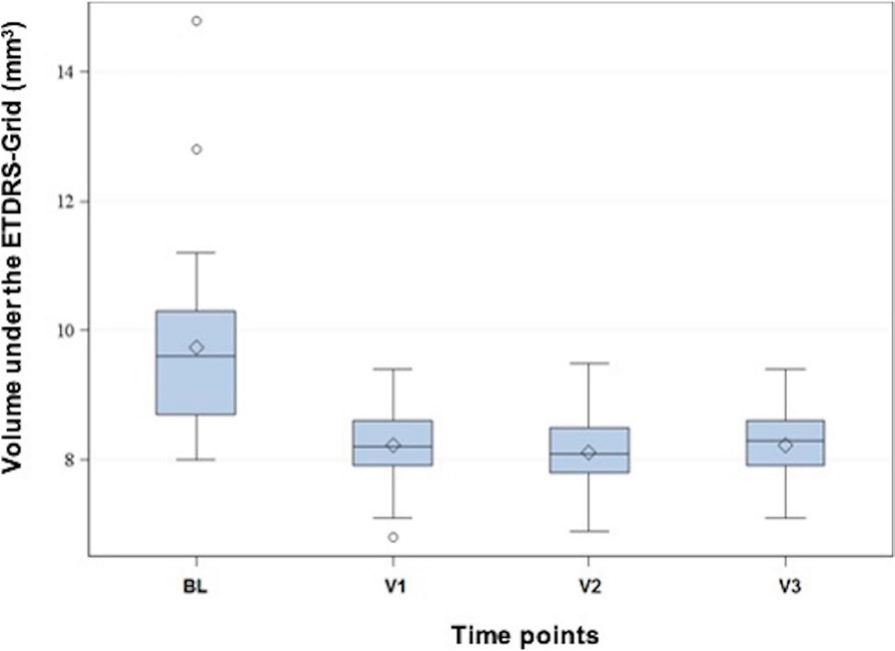
Volume measured in OCT under the ETDRS grid at the different time points. On the ordinate is the volume under the ETDRS grid (mm^3^), the different times points are plotted on the abscissa. The diamond indicates the mean, and the horizontal line shows the median of the volume. The gray box in the Tukey diagram marks the range of values from the first to the third quartile, the whiskers have a length of 2 times the interquartile range. Measured values outside this range are shown as outliers in the diagram by means of a circle. ETDRS: early treatment diabetic retinopathy study; BL: baseline; V: visit

Figure 2 shows the volume measured in OCT under the ETDRS grid at the time points BL and V1 to V3. The median volume at BL of 9.6 mm^3^ dropped to 8.2 mm^3^ at V1, 8.1 mm^3^ at V2, and 8.3 mm^3^ at V3. The changes from V1 to V3 were statistically significant compared to BL (*p* < 0.05).

Central retinal subfield thickness at the time points BL and V1 to V3 was investigated. The initial median thickness of 397 µm at BL decreased to 264 µm at V1, to 236 µm at V2 and to 239 µm at V3. At time points V1 to V3, the differences compared to BL were statistically significant (*p* < 0.05).

**Fig. 3:**
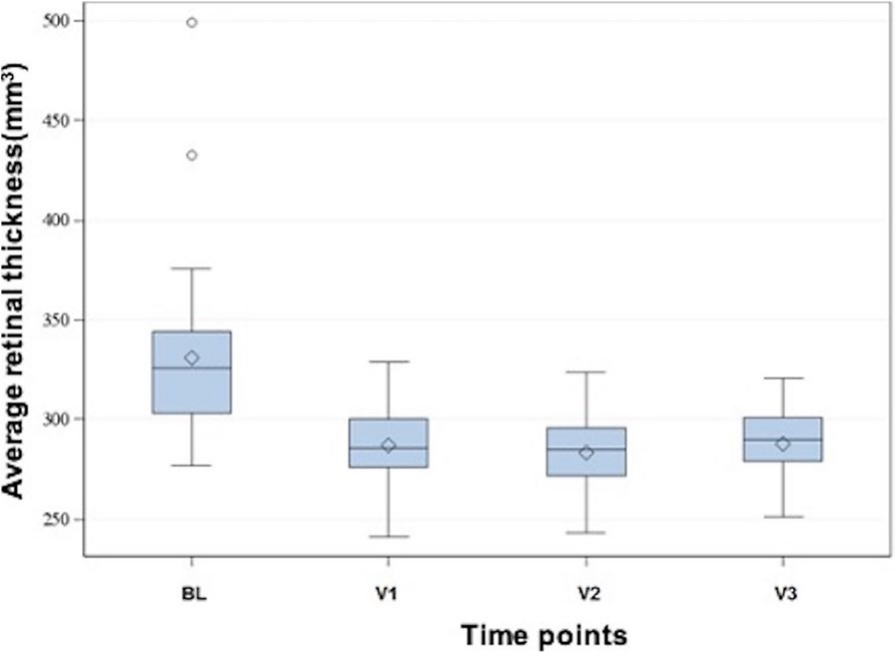
Average retinal thickness measured by OCT in the whole cube at the different time points. The ordinate shows the average thickness of the retina in the whole cube (µm), the abscissa demonstrates the different time points. The diamond indicates the mean, the horizontal line the median of the visual acuity. The gray box in the Tukey diagram marks the range of values from the first to the third quartile, the whiskers have a length of 2 times the interquartile range. Measured values outside of this range are shown as outliers in the diagram by means of a circle. BL: Baseline; V: Visit

The average thickness of the retina in the entire data cube of the OCT at the time points BL and V1 to V3 is displayed in Fig. 3. The average cube thickness of 326 µm in BL decreased over time to 286 µm in V1, 285 µm in V2, and 290 µm in V3. Significant differences were found for V1 to V3 compared to BL (*p* < 0.05).

**Fig. 4:**
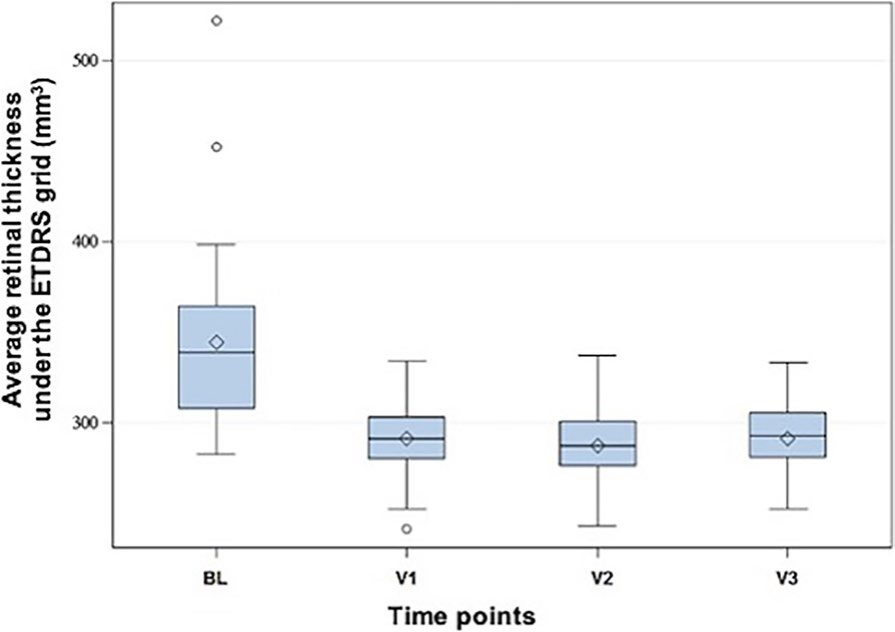
Average retinal thickness under the ETDRS grid measured by OCT at the different time points. The ordinate shows the average thickness of the retina under the ETDRS grid (µm), the abscissa indicates the different time points. The gray box in the Tukey diagram marks the range of values from the first to the third quartile, the whiskers have a length of 2 times the interquartile range. Measured values outside this range are shown as outliers in the diagram by means of a circle. ETDRS: early treatment diabetic retinopathy study; BL: baseline; V: visit

Figure 4 shows the average thickness of the retina under the ETDRS grid at the time points BL and V1 to V3. While the median of the average retinal thickness at time point BL was 339 µm, this dropped to 291 µm at time point V1, 287 µm at V2 and 293 µm at V3. The changes at time points V1, V2 and V3 compared to BL were statistically significant (*p* < 0.05).

No correlation between BCVA at BL and various parameters of the patient cohort was found (Table 1). No correlation between BCVA at V3 and various parameters of the patient collective was found either (Table 2).

**Table 1:** Spearman correlation analysis to test for a possible relationship between best-corrected visual acuity before laser coagulation and various parameters collected

Parameter	Spearman correlation coefficient	*p*-value	Number of eyes evaluated
Age	0.33401	0.0145	53
Duration until ST-LP	0.00119	0.9933	52
Visual acuity at last visit	0.55745	< 0.0001	50
Central ret. thickness BL	-0.07898	0.6281	40
Volume in OCT cube BL	-0.19257	0.2339	40
Average ret. thickness BL	-0.19466	0.2287	40
Volume under ETDRS grid in OCT BL	-0.11504	0.4797	40
Average ret. thickness under ETDRS grid BL	-0.10641	0.5134	40
Central ret. thickness V1	-0.46029	0.0054	35
Volume in OCT cube V1	-0.26847	0.1189	35
Average ret. thickness V1	-0.25342	0.1419	35
Volume under ETDRS grid in OCT V1	-0.25565	0.1383	35
Average ret. thickness under ETDRS grid V1	-0.26195	0.1285	35
Central ret. thickness V2	-0.32355	0.1321	23
Volume in OCT cube V2	-0.11969	0.5865	23
Average ret. thickness V2	-0.11227	0.6100	23
Volume under ETDRS grid in OCT V2	-0.15675	0.4751	23
Average ret. thickness under ETDRS grid V2	-0.14217	0.5176	23
Central ret. thickness V3	0.30556	0.2881	14
Volume in OCT cube V3	0.39540	0.1617	14
Average ret. thickness V3	0.40467	0.1512	14
Volume under ETDRS grid in OCT V3	0.38223	0.1774	14
Average ret. thickness under ETDRS grid V3	0.40491	0.1510	14

*logMAR* logarithmic reciprocal of visual acuity, *ST-LP* subthreshold laser photocoagulation, *ret.* Retinal, *BL* baseline, *OCT* optical coherence tomography, *ETDRS* early treatment diabetic retinopathy study, *V* visit, *p*-*value* excess probability

**Table 2:** Spearman correlation analysis to test for a possible relationship between best-corrected visual acuity at the last visit and various parameters collected

Parameter	Spearman correlation coefficient	*p*-value	Number of eyes evaluated
Age	0.38859	0.0048	51
Period until ST-LP	0.26815	0.0571	51
Central ret. thickness BL	-0.03736	0.8238	38
Volume in OCT cube BL	-0.17294	0.2991	38
average ret. thickness BL	-0.17389	0.2964	38
Volume under ETDRS grid in OCT BL	-0.08268	0.6217	38
Average ret. thickness under ETDRS grid BL	-0.08924	0.5942	38
central ret. thickness V1	-0.04074	0.8162	35
Volume in OCT cube V1	-0.31850	0.0622	35
Average ret. thickness V1	-0.32014	0.0608	35
Volume under ETDRS grid in OCT V1	-0.26997	0.1168	35
Average ret. thickness under ETDRS grid V1	-0.25693	0.1362	35
central ret. thickness V2	-0.27965	0.1962	23
Volume in OCT cube V2	-0.20368	0.3513	23
average ret. thickness V2	-0.18358	0.4018	23
Volume under ETDRS grid in OCT V2	-0.19012	0.3849	23
Average ret. thickness under ETDRS grid V2	-0.19705	0.3675	23
central ret. thickness V3	0.31746	0.2489	15
Volume in OCT cube V3	0.08238	0.7704	15
average ret. thickness V3	0.10620	0.7064	15
Volume under ETDRS grid in OCT V3	0.07656	0.7862	15
average ret. thickness under ETDRS grid V3	0.10012	0.7226	15

*logMAR* logarithmic reciprocal of visual acuity, *ST-LP* subthreshold laser photocoagulation, *ret.* Retinal, *BL* baseline, *OCT* optical coherence tomography, *ETDRS* early treatment diabetic retinopathy study, *V* visit, *p-value* excess probability

## Discussion

All macular parameters measured by OCT—volume in the OCT cube, volume under the ETDRS grid, central retinal subfield thickness, average retinal thickness and average retinal thickness under the ETDRS grid – developed very similarly after ST-LP. At time points V1 to V3, i.e., 1 month, 2 months, and 3 months after ST-LP, we found a statistically significant decrease in measured thickness or volume. This is in accordance with our previously published findings in this cohort, which demonstrated that SRF could no longer be detected in two thirds of the patients at V3 [10].

The distribution of the measured values is remarkable. Indeed, they are very similar for macular volume, macular volume under the ETDRS grid, average retinal thickness and average retinal thickness under the ETDRS grid: before the ST-LP at BL a larger box is shown (50% of the measured values), which becomes smaller after laser treatment at time points V1 to V3. However, the whiskers indicate a roughly constant scatter of the measured values before and after laser coagulation. Measurement outliers, i.e., extremes in macular volume and average retinal thickness, are no longer found at time points V1 to V3. This means that the ST-LP by its mechanism of action discussed below seems to be able to exert its effect independently of the preexisting retinal thickness and resorption of SRF, which requires an intact RPE.

Compared to the aforementioned parameters, the central retinal subfield thickness showed a somewhat different behavior. The range of variation of half of the measured values (gray box), as well as of the minimum and maximum values, was significantly larger. Again, after ST-LP, there was a marked decrease in central retinal subfield thickness with a significant reduction in the range of variation of the measured values from V1 to V2. In V3, the range of variation increased again with a stronger measurement outlier than in V2. One possible reason could be an increasing accumulation of SRF in some patients 3 months after ST-LP. If so, the central retinal subfield thickness parameter might be a better tool to detect renewed disease activity earlier than average retinal thickness measurement or volume measurement. On the other hand, it must be stated that the median retinal subfield thickness remained clinically stable from V2 to V3. The different behavior of central retinal subfield thickness compared with the other parameters could be due to foveal depression, because there is a greater percentage change in retinal thickness for the same volume increase than for extrafoveal location. In addition, involvement of the central retina is a characteristic of CSCR, so the fovea is likely to be involved in most cases [11]. This study provides no evidence that examination of retinal thickness or retinal volume in the whole OCT scan is inferior or superior to measurement under the ETDRS grid. This is relevant in light of the multiple display and calculation capabilities of modern OCT software. However, care should be taken to control with the same modality in follow-up examinations.

Our study shows that there was a clear, statistically significant decrease in retinal thickness and retinal volume after ST-LP. A renewed increase in disease activity in terms of retinal thickness or volume was not seen before the 3rd month after ST-LP.

Spearman correlation analysis (SCA) was performed with respect to BCVA before ST-LP and BCVA at last visit to identify possible correlations with selected parameters. In this work, a Spearman correlation coefficient of > 0.8 or < -0.8 was required to detect a strong association between two characteristic values.

The SCA does not show a correlation between patient age and visual acuity before the ST-LP and after the last visit. Although there is evidence in the literature that older age is more likely to be associated with an atypical chronic course, the heterogeneous patient population presented here may be too small to demonstrate an association [12, 13].

With a Spearman correlation coefficient of 0.56, there could be a moderate association between BCVA at last visit and BCVA at BL. A *p*-value of < 0.0001 with a case number of 50 patients after all supports this assessment. This possible association seems plausible, as poorer baseline visual acuity could be associated with structural retinal damage.

In the present data, no correlation could be established between disease duration and visual acuity before treatment and at the last visit. This is surprising at first, because according to pathophysiological considerations, if SRF persists for a longer time and thus the NR is detached from the retinal pigment epithelium and the blood-bearing choroid, which supplies oxygen to the outer retinal layers, damage to the retina with visual loss is to be expected. Similarly, persistent SRF and disease progression over 5 years with cystoid degeneration of the retina have been shown to be associated with poor BCVA [14, 15]. It is conceivable that the disease duration in the present study, with a median of 9 weeks of current symptoms, is too short to damage the retina and thus allow good visual rehabilitation. However, 19% of the patients had already received physical therapy for the treatment of CSCR in the past and 80% of the patients had a relapse or a duration of disease longer than 6 months. This very heterogeneous composition of the patient population could mask a possible relationship between visual acuity and disease duration.

Furthermore, there no correlation between BCVA and different thickness and volume measurements of the macula before ST-LP and at the last visit was seen. Again, it can be assumed that a more pronounced detachment of the NR leads to a greater reduction of visual acuity before therapy initiation and a greater retinal damage with worse visual acuity at the last visit than a slight detachment at most. Other possible factors influencing visual acuity, such as duration of detachment and possibly previous retinal damage due to a past disease episode, could be confounding variables masking a possible relationship in this study.

We conclude that after ST-LP, there was a rapid decrease in retinal thickness and retinal volume. Central retinal subfield thickness measured by OCT may be a more sensitive measure than mean retinal thickness or macular volume for early detection of disease recurrence.

Age, duration of the disease, different measurement parameters of macular thickness and macular volume were not correlated to BCVA before and after ST-LP.

## Data Availability

The datasets generated and/or analyzed during the current study are not publicly available to avoid infringement of copyright but are available from the corresponding author on reasonable request.

## References

[1] Manayath GJ, Ranjan R, Karandikar SS, Shah VS, Saravanan VR, Narendran V (2018). Central serous chorioretinopathy: current update on management. Oman J Ophthalmol.

[2] Spaide RF, Goldbaum M, Wong DW, Tang KC, Iida T (2003). Serous detachment of the retina. Retina.

[3] Sheth J, Anantharaman G, Chandra S, Sivaprasad S (2018). "Double-layer sign" on spectral domain optical coherence tomography in pachychoroid spectrum disease. Indian J Ophthalmol.

[4] Nicholson B, Noble J, Forooghian F, Meyerle C (2013). Central serous chorioretinopathy: update on pathophysiology and treatment. Surv Ophthalmol.

[5] Bousquet E, Provost J, Zola M, Spaide RF, Mehanna C, Behar-Cohen F (2021). Mid-Phase Hyperfluorescent Plaques Seen on Indocyanine Green Angiography in Patients with Central Serous Chorioretinopathy. J Clin Med.

[6] Yannuzzi LA (2010). Central serous chorioretinopathy: a personal perspective. Am J Ophthalmol.

[7] Iida T, Kishi S, Hagimura N, Shimizu K (1999). Persistent and bilateral choroidal vascular abnormalities in central serous chorioretinopathy. Retina.

[8] Scheider A, Nasemann JE, Lund OE (1993). Fluorescein and indocyanine green angiographies of central serous choroidopathy by scanning laser ophthalmoscopy. Am J Ophthalmol.

[9] Hanumunthadu D, Tan ACS, Singh SR, Sahu NK, Chhablani J (2018). Management of chronic central serous chorioretinopathy. Indian J Ophthalmol.

[10] Enders C, Lang GE, Mayer B, Werner JU. Central Serous Chorioretinopathy: Morphological and Functional Outcome after Subthreshold Thermal Laser Coagulation with a Frequency-Doubled Nd:YAG Continuous-Wave Laser. Ophthalmologica. 2021. 10.1159/000519234. Epub ahead of print. 10.1159/000519234 34517369

[11] Daruich A, Matet A, Dirani A, Bousquet E, Zhao M, Farman N, Jaisser F, Behar-Cohen F (2015). Central serous chorioretinopathy: recent findings and new physiopathology hypothesis. Prog Retin Eye Res.

[12] Lafaut BA, Salati C, Priem H, De Laey JJ (1998). Indocyanine green angiography is of value for the diagnosis of chronic central serous chorioretinopathy in elderly patients. Graefes Arch Clin Exp Ophthalmol.

[13] Bülow N (1978). The process of wound healing of the avascular outer layers of the retina. Light- and electron microscopic studies on laser lesions of monkey eyes. Acta Ophthalmol Suppl.

[14] Loo RH, Scott IU, Flynn HW, Gass JD, Murray TG, Lewis ML, Rosenfeld PJ, Smiddy WE (2002). Factors associated with reduced visual acuity during long-term follow-up of patients with idiopathic central serous chorioretinopathy. Retina.

[15] Iida T, Yannuzzi LA, Spaide RF, Borodoker N, Carvalho CA, Negrao S (2003). Cystoid macular degeneration in chronic central serous chorioretinopathy. Retina.

